# Chitosan Nanoparticles Encapsulating Oregano Oil: Effects on In Vitro Ruminal Fermentation from Goat Rumen Fluid

**DOI:** 10.3390/ani15152261

**Published:** 2025-08-01

**Authors:** Gerardo Méndez-Zamora, Jorge R. Kawas, Sara Paola Hernández-Martínez, Gustavo Sobrevilla-Hernández, Sugey Ramona Sinagawa-García, Daniela S. Rico-Costilla, Jocelyn Cyan López-Puga

**Affiliations:** 1 Facultad de Agronomía, Universidad Autónoma de Nuevo León, Francisco Villa s/n, Ex-Hacienda El Canadá, General Escobedo 66050, Nuevo León, Mexico; gerardo.mendezzm@uanl.edu.mx (G.M.-Z.); jorge.kawasgr@uanl.edu.mx (J.R.K.); sara.hernandezma@uanl.edu.mx (S.P.H.-M.); sugey.sinagawagr@uanl.edu.mx (S.R.S.-G.); 2 MNA de México, Carretera Huinalá-Villa Juárez, km 59.6, Juárez 67250, Nuevo León, Mexico; gsobrevillah@outlook.com; 3 Facultad de Medicina Veterinaria y Zootecnia, Universidad Autónoma de Nuevo León, Francisco Villa s/n, Ex-Hacienda El Canadá, General Escobedo 66050, Nuevo León, Mexico; daniela.ricocst@uanl.edu.mx

**Keywords:** goats, essential oils, digestion, gas production, ammonia

## Abstract

This study evaluated the effects of liquid oregano oil, chitosan nanoparticles with encapsulated liquid oregano oil, and a negative control of empty chitosan nanoparticles on in vitro ruminal fermentation. Three Boer goats were used as ruminal fluid donors, fed with a formulated ration for 21 d for inoculum adaptation. Treatments tested on in vitro assays were diet without oregano oil or nanoparticles (CON); diet with 100 ppm of oregano oil in nanoparticles (100N); diet with 300 ppm of liquid oregano oil (300L); diet with 300 ppm of oregano oil in nanoparticles (300N); and diet with 300 ppm of empty nanoparticles (300CHN). The variables studied were in vitro dry matter digestibility (ivDMD), in vitro neutral detergent fiber digestibility (ivNDFD_om_), total gas production (TGP), ammonia nitrogen concentration (NH_3_), and pH. The experimental design was a randomized complete block design. Linear and quadratic regressions were used to identify dependence and inflection points. The ivDMD increased at 12, 36, and 48 h, with 300N and with 300L exhibiting increased ivNDFD_om_ at 36 h. Ruminal pH was highest (*p* < 0.05) with 300CHN at 36 h. For first-order regression analysis of TGP, coefficients (β) were highest (*p* < 0.05) for 300N. In conclusion, 300N increased ruminal fermentation in vitro, as reflected by increases in ivDMD, ivNDFD_om_, and TGP.

## Simple Summary

One of the most promising alternatives to the use of prophylactic antibiotics in production is the use of essential oils; the most studied route of administration has been orally through feed. In Mexico, one of the most studied essential oils is oregano essential oil (*Lippia berlandieri* Shauer). However, its use faces several problems, such as preserving the bioactive properties of the essential oil during its passage through the rumen. In addition, its use has been studied mainly in non-ruminants, so characterizing its effects in the rumen is necessary to standardize doses. One way to preserve bioactive properties is through encapsulation, so this work evaluated nanoencapsulated oregano essential oil on in vitro fermentation using goat ruminal fluid. The treatments used were diet without oregano oil or nanoparticles (CON); diet with 100 ppm of oregano oil in nanoparticles (100N); diet with 300 ppm of liquid oregano oil (300L); diet with 300 ppm of oregano oil in nanoparticles (300N); and diet with 300 ppm of empty nanoparticles (300CHN). The results indicate that the treatment of 100N had similar effects to 300L, proving that minor doses of oregano nanoparticles can be used to reduce doses in the rumen.

## 1. Introduction

The antimicrobial activity of essential oils (EOs) extracted from aromatic plants [1] has been of strategic interest because their volatile compounds help serve as a replacement for the use of growth promoters in ruminants due to their selective antibacterial activity and inhibition of protein degradation in the rumen, thus showing increases in intestinal bioavailability of amino acids [2]. The addition of EO to small ruminant diets provides bioactive volatile and lipophilic phenolic compounds that improve dairy product qualities [3] by benefiting ruminal fermentation and productive performance, improving the functionality of dairy products that benefit consumer health [4].

Oregano essential oil (OEO) has been investigated in ruminal fermentation and in vitro digestibility [5]. The OEO contains thymol and carvacrol as active ingredients, which have been evaluated at different doses and have exhibited effects on in vitro ruminal fermentation, like altering pH, making changes in bacteria populations, altering ammonia, methane, and total gas production (TGP) [6,7,8,9,10,11]. Results from studies on OEO are contrasting in terms of dosage: for example, 200 mgL^−1^ of oregano in ruminal fluid seems to lower fiber digestibility and not produce changes in gas production at 24 h [2], but at 1.0 mmol/L, it increases TGP at 16 h, ammonia and volatile fatty acids (VFAs) [8], which contrasts with other authors [9], who reports that at a dose of 0.10 g/L, microbial populations decrease and butyrate increases, while at 30 mg/L methane, ammonia and VFAs were not affected. Despite these contrasting results, OEO had remarkable benefits in goat rumen fermentation in the in vivo trials when the whole plant was added, increasing the antioxidant system in the animal through antioxidant enzyme activation, and this enhanced the antioxidant capacity of milk from the supplemented goats [12]. Hence, the problematic factor in the use of OEO on in vitro and in vivo studies, there is the need to optimize the inclusion of OEO in diets to reduce methane production without altering feed digestion, rumen fermentation, productivity, and meat or milk quality [11].

The OEO can be added directly into animal feed [13], but essential oils can also be nanencapsulated before being added [14]. In this process of nanoencapsulation, a barrier is formed between the interior content and the exterior environment by the wall of the nanocapsule, excluding external chemical and physical reactions and influences [15]. Essential oils have great potential as feed additives, although they are unstable under certain conditions of exposure to oxygen, light, humidity, and heat, affecting their chemical composition. Hence, nanoencapsulation of OEO as a feed supplement could be a viable option to maintain biological and functional characteristics [15] and lower doses of a drug or bioactive component [16]. In recent years, the development of biodegradable nanoparticles has been studied as an effective lipophilic bioactive food component delivery system [14]. Similarly, chitosan (CH) is a biopolymer used to encapsulate bioactive compounds due to its biocompatibility, low toxicity, and biodegradability [14]. Thus, liquid oregano oil (*Lippia berlandieri* Schauer) may have different effects on rumen fermentation than nanoparticle-encapsulated oregano oil (OON).

Oregano oil and nanoparticles in dairy goat rations have been given little attention, and it has not been previously tested whether nanoparticles used in the rumen can reduce the dosage of OEO. Therefore, this study was conducted to evaluate the effects of liquid oregano oil and liquid oregano oil encapsulated in chitosan nanoparticles on in vitro ruminal fermentation from goat rumen fluid. The study hypothesis was that lower doses of oregano oil encapsulated in chitosan nanoparticles would have similar effects on in vitro ruminal fermentation from goat rumen fluid compared to higher doses of liquid oregano oil.

## 2. Materials and Methods

### 2.1. Synthesis and Characterization of Chitosan Nanoparticles

The synthesis and characterization of nanoparticles were carried out in the Metabolismo y Nutrición Animal Laboratory in Facultad de Medicina Veterinaria y Zooteecnia, Universidad Autónoma de Nuevo Léon, General Escobedo, México, and the Nanotechnology Department of Inmunology Laboratory in Facultad de Biología, Universidad Autónoma de Nuevo León, San Nicolás de los Garza, Nuevo León, México. Oregano oil was extracted by steam distillation at 60 °C from the leaves of *Lippia berlandieri* Schauer and was composed of 65.20% carvacrol, 10.99% p-Cymene, 10.26% thymol, 2.71% caryophyllene, and 8.81% other compounds. Chitosan nanoparticles (CHNs) and chitosan nanoparticle-encapsulated liquid oregano oil were prepared according to the method described by Hosseini et al. [14] with slight modifications specified subsequently. For CHNs, 1% chitosan (CH; Sigma-Aldrich^®^, Burlington, MA, USA) solution in 0.1 M acetic acid was prepared by adding 2% Tween 80 (Sigma-Aldrich^®^, Saint Louis, MO, USA) and stirring for 2 h until a homogeneous solution was obtained. Then, 2 mL of sodium tripolyphosphate (TPP; Sigma-Aldrich^®^, Saint Louis, MO, USA) was added at 2 mg/mL and stirred for 40 min. The CHNs were recovered by centrifugation at 4500× *g* for 20 min at 22 °C (Eppendorf 5804r, Oldenburg, Lower Saxony, Germany), and the supernatant was removed. The same procedure was followed for OON, except for an emulsion that was made before adding TPP. The emulsion was prepared separately, dissolving 0.5 mg of OEO in 1 mL of 100% ethanol (CTR Scientific; Monterrey, N.L., Mexico), then slowly added into 10 mL of the aqueous CH solution during homogenization at 500 rpm. The suspension of OON was centrifuged at 4500× *g* for 20 min at 22 °C (Eppendorf 5804r), and the supernatant was removed. CHN and OON centrifugation pellets were lyophilized at −35 °C using a FreeZone 4.5 lyophilizer (LABCONCO; Kansas City, MO, USA). Particle size was measured by dynamic light scattering (DLS) (Zetasizer ZS90-Nano; Malvern Instruments, Malvern, Worcs., UK). An image of chitosan nanoparticles with oregano oil encapsulated were taken using a scanning electron microscope at 2.00 μm.

### 2.2. Experimental Diet and Chemical Composition

An isoproteic–isoenergetic diet was formulated according to NRC [17] requirements. The diet was prepared at MNA de Mexico S.A. de C.V. (Juárez, Nuevo León, Mexico) using an industrial mixer. Chemical analysis was performed according to AOAC [18] procedures (Table 1).

### 2.3. Experimental Animals

In vitro fermentation was performed with pooled ruminal fluid from three 3-year-old lactating Boer goats. Goats were fed an experimental diet (Table 1) for a 21-day adaptation period. On day 22, two hours after evening feeding, ruminal fluid extraction was performed using an orogastric tube under animal welfare guidelines of the Official Mexican Standard NOM-062-ZOO [19]. The study was approved by the Animal Welfare Committee of the Facultad de Medicina Veterinaria y Zootecnia, Universidad Autónoma de Nuevo León (09/2020 approval number). Goats were housed in an experimental area at the Metabolismo y Nutrición Animal Laboratory in the Facultad de Medicina Veterinaria y Zooteecnia, Universidad Autónoma de Nuevo León, General Escobedo, México, where all in vitro tests were conducted.

### 2.4. In Vitro Digestibility

In vitro digestibility was examined in a Daisy II incubator (ANKOM Technology; Macedon, NY, USA) according to method 3 of ANKOM Technology [20]. ANKOM F57 fiber filter bags (5 × 5 cm; pore size 25 µ) were used in this phase, which were rinsed with acetone, labeled, and weighed. Then, 1 kg of total mixed ration (TMR) was ground in a mill (Thomas Scientific Wiley Model 4, Swedesboro, NJ, USA) for 5 min. Then, ground TMR was mixed with established treatments using liquid oregano oil encapsulated in chitosan nanoparticles (OON) and control negatives of only diet and empty chitosan nanoparticles (CHNs). Treatments were diet without oregano oil or nanoparticles (CON); diet with 100 ppm of oregano oil in nanoparticles (100N); diet with 300 ppm of liquid oregano oil (300L); diet with 300 ppm of oregano oil in nanoparticles (300N); and diet with 300 ppm of empty nanoparticles (300CHN). Treatment doses (100 and 300 ppm) in nanoparticles and CHNs were estimated and balanced to the actual quantity for a correct dose according to liquid oregano oil content. They were established to monitor changes between a lower and a higher dose of nanoparticles or compared to liquid oregano oil. Next, 0.50 g of the treatments were placed into ANKOM F57 bags, weights were recorded, and the bags were sealed. Sample bags were placed inside ANKOM Daisy II jars (ANKOM Technology), and the ANKOM F57 bags were distributed between the two sides of the jars; an empty control bag was also included, equivalent to the correction factor. Each jar contained an experimental treatment. A buffer solution (1600 mL) that simulates animal saliva and approximately 400 mL of ruminal fluid from the goats (133.33 mL of ruminal fluid from each goat) were added to each jar [20]. Ruminal fluid was filtered through four layers of sterile absorbent gauze (Quirmex, Zacatepec, Puebla, Mexico) before being mixed with buffer solution [20]. At the same time, a stream of CO_2_ was supplied for maintenance and development of anaerobic bacteria. The ruminal fluid digestion jar assemblies were introduced into the Daisy II Incubator. In vitro fermentation was carried out for 48 h at 39.5 ± 0.5 °C. After incubation, the outsides of the bags were rinsed with tap water, and the bags were dried for 48 h in a drying oven at 60 °C. Weights of dried bags were recorded to calculate in vitro dry matter digestibility (ivDMD; Equation (1)). Afterward, ANKOM F57 bags were placed into the ANKOM 2000 Fiber Analyzer (ANKOM Technology) to determine the in vitro digestibility of neutral detergent fiber (ivNDFD) and α-amylase activity [21]. After determination of ivNDFD, bag weights were recorded, and then ash content was determined after drying at 550 °C for 2 h to adjust the ivNDFD based on organic matter (_om_). Thus, the ivNDFD was estimated considering the organic matter in its calculation (ivNDF_om_). This adjustment was made (Equation (2)) according to Mertens [22] and Udén et al. [23]. For the ivDMD and ivNDFD_om_ variables, each treatment was analyzed in triplicate (3 bags/treatment/time) in a digestion jar, and 3 jars were used in total, where each jar was considered as a block in the data analysis (n = 225; ANKOM F57 total bags and 3 blank bags). To facilitate the entry and exit of the bags to ruminal fluid at the same time, bags were sewn at the top of the bag before the sealing line with a nylon thread ring in batches by time, to be removed simultaneously at 0, 12, 24, 36, and 48 h. Bags were previously labeled by time, repetition, treatment, and jar (block).

(1) $$\%ivDMD=100-\left[\frac{\left(Bag\;weight\;after\;in\;vitro\;incubation-\left(Bag\;tare\;weight\times Correction\;bag\right)\right)}{Sample\;weight\times Dry\;matter}\right]\times 100$$

(2) $$\%{ivNDFD}_{om}=\left[\frac{Loss\;of\;weight\;on\;ignition\;of\;bag\;and\;fiber\;residue-(Bag\;tare\;weight\times Ash\;corrected\;blank\;bag)}{Samplewight\times DM}\right]\times 100$$

### 2.5. Total Gas Production (TGP), pH, and Ammonia (NH_3_)

Total gas production was measured at 5 min, 3, 6, 9, 12, 15, 18, 21, and 24 h using the ANKOM gas production system [24]. A jar from each module of the gas production system was assigned per treatment, and three readings of TGP measurements from the computer program by jar were taken each time. This experiment was replicated six times to consider each experiment repetition as a block in data analysis and the jar as the experimental unit. Reads of TGP by jar were taken in triplicate (n = 3 lectures/jar/time/experimental repetition (block) = 810 TGP reads in total). Ground TMR (0.25 g) from each treatment was weighed and added to a jar. Then, 20 mL of ruminal fluid was added to each jar, which was placed in a water bath at 39 °C. Constantly, CO_2_ was streamed into the jar during the handling of rumen fluid for anaerobic bacteria maintenance. Ground TMR and ruminal fluid were added to jars according to the ANKOM Gas Production System instructions [24]. Once fermentation was complete and data were collected, gas pressure measured during the evaluation was converted to moles of gas produced using the ideal gas law (Equation (3)) and converted to milliliters of gas produced using Avogadro’s law (Equation (4)), as follows:

(3) $$n\;=\;p\left(\frac{V}{RT}\right)\times 100$$

where

n = Total gas produced (mol);
p = Pressure (kPa);
V = Head-space volume in bottle (L);
T = Kelvin temperature (°K);
R = Gas constant (8.314472 L·kPa·K^−1^ mol^−1^).

(4) Total gas production (mL)=n × 22.4 × 1000

Ruminal fluid pH and NH_3_ were determined at 0, 3, 6, 12, and 24 h in triplicate (n = 3 repetitions/treatment/time = 75 samples in total) after incubating the samples in a water bath at 39 °C following the Broderick and Kang [25] method. Initially, ruminal fluid samples in 50 mL Falcon tubes (Corning Incorporated, Corning, NY, USA), at a concentration of 20 mg of DM/mL of ruminal fluid, were held in a water bath at 39 °C for 0, 3, 6, 12, and 24 h. After incubation, pH was measured by inserting a UB-10 potentiometer (Denver Instrument, Bohemia, NY, USA) into the ruminal fluid sample tubes at the end of the incubation period before centrifuging for NH_3_. Next, NH_3_ was measured by spectrophotometry according to Broderick and Kang [25] with slight modifications. Samples of ruminal fluid were centrifuged at 12,000× *g* for 20 min (Hermle Z 300, Labnet, Edison, NJ, USA). The supernatants were used to determine the NH_3_ concentration, using 40 μL of ruminal fluid. Subsequently, 2.5 mL of sodium phenol-nitroprusside reagent and 2.0 mL of alkaline hypochlorite reagent were added for indophenol formation, whose absorbance is proportional to the NH_3_-N concentration in the sample [26]. From each tube, 300 μL was transferred to the wells of a 96-well microtiter plate (Thermo Fisher Scientific, Waltham, MA, USA) to measure absorbance at a wavelength of 630 nm (ChroMate^®^; Awareness Technology, Inc.; Palm City, FL, USA).

### 2.6. Statistical Analysis

Data from ivDMD and ivNDFD_om_ were analyzed using the general linear model (GLM) to evaluate the effect of treatment (T_i_), block 1 (jar; β_j_), block 2 (time; δ_j_) and treatment/time interaction [(T δ)_ik_] considering the following statistical model: y_ijk_ = μ + T_i_ + β_j_ + δ_k_ + (T δ)_ik_ + ε_ijkl_. Data from TGP were analyzed using the general linear model (GLM) to evaluate the effect of treatment (T_i_), block 1 (replication; β_j_), block 2 (time; δ_k_), and treatment/time interaction [(T δ)_ik_] considering the following statistical model: y_ijk_ = μ + T_i_ + β_j_ + δ_k_ + (Tδ)_ij_ + ε_ijk_. Data from pH and NH_3_ were analyzed using a general linear model (GLM) to evaluate the effect of treatment (T_i_) and time (δ_j_) and their interaction [(T δ)_ij_] considering the following statistical model: y_ij_ = μ + T_i_ + δ _j_ + (T δ)_ij_ + ε_ijk_. When H_0_ was rejected (*p* ≤ 0.05) for fixed effects and interaction, means comparison was performed using comparison instruction (adjusted Tukey). First- and second-order simple linear regression was performed to evaluate the dependence of y as a function of time (*X*) for each treatment, considering the following statistical model: y = β_0_ + β_1_*X*_i_ + E_i_. Likewise, the quadratic regression model included β_11_X^2^_11_. Afterward, since ivDMD and ivNDFD_om_ were performed with nine replications per treatment, nine linear regression models (nine regression coefficients) were obtained per treatment, and for TGP, pH, and NH_3_, six linear regression models were obtained (regression coefficients). Next, an analysis of variance was performed with the GLM instruction by coefficient regression of all treatments to obtain the analysis of β_1_ and β_11_. When H_0_ was rejected (*p* ≤ 0.05), a comparison of means was made using the Tukey test. A polynomial regression analysis of total gas production per treatment over time was made, where the response variable (Y) in the data analysis was TGP and the predictor (X) was time. This procedure was repeated per treatment over time to identify linear, quadratic, and cubic slopes. All data analyses were performed with Minitab 17^®^ (Sate College, PA, USA) [27] software.

## 3. Results

### 3.1. Nanoparticle Size

**Figure 1:**
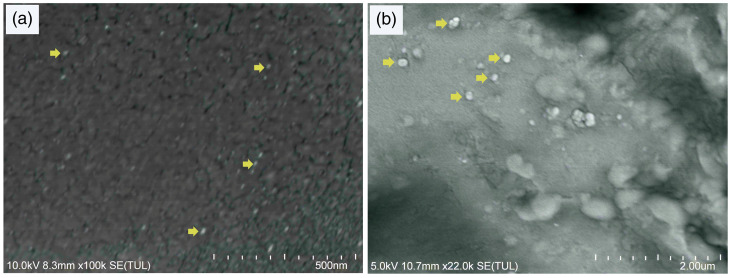
Scanning electron microscope images of (**a**) CHNs and (**b**) OON. Yellow arrows show examples to identify nanoparticles.

Chitosan nanoparticles encapsulating oregano oil (OON) had a nanoparticle size of 332.95 nm, while chitosan nanoparticles without OEO (CHNs) were 3.39 nm. In Figure 1, an image of OON is shown.

### 3.2. In Vitro Dry Matter Digestibility (ivDMD)

Table 2 presents the in vitro dry matter digestibility (ivDMD) of the treatments fermented for 48 h in goat ruminal fluid. At 12 h, the treatment with 300N had the highest (*p* < 0.05) ivDMD, 5.61% higher compared to CON. At 36 h, treatment with 300N resulted in a better ivDMD by 3.91% compared to 300L. At 48 h, 300N had higher ivDMD by 3.89% compared to CON.

### 3.3. In Vitro Neutral Detergent Fiber Digestibility on an Organic Matter Basis (ivNDFD_om)_

For ivNDFD_om_, a significant effect was found in the time principal effect (Table 2). At 36 h, 300L had a higher ivNDFD_om_ (*p* < 0.05) by 7.04% compared to 300N. For 300CHN, the difference in the ivNDFD_om_ was 1.40% lower than 300L.

### 3.4. First- and Second-Order Linear Regression Analysis of ivDMD and ivNDFD_om_

First-order regression analysis for the ivDMD and ivNDFD_om_ of OEO treatments indicated that these variables were time-dependent (β_1_; *p* < 0.05), obtaining a model fit of 81.90% (CON) to 83.60% (300L) for ivDMD, and 42.36% (300N) to 71.85% (300L) for ivNDFD_om_ (Table 3). First-order slopes were not different (*p* > 0.05) between treatments.

Results of second-order linear regression analysis (β_1_ and β_11_; quadratic) for treatments showed that ivDMD was dependent (*p* < 0.05) on time (β_1_ and β_11_), as well as ivNDFD_om_ (β_1_; *p* < 0.05) (Table 3). Likewise, quadratic regression analysis indicated an adjustment from 96.79% (100N) to 97.45% (CON) for ivDMD. However, for ivNDFD_om_, the adjustment increased (42.73% in 300N and 72.05% in 300L).

### 3.5. Total Gas Production

The principal effects of treatment and time were significant (*p* < 0.05). The treatment of 300N had the highest (*p* < 0.05) TGP in all the treatments, as shown in Table 4.

**Figure 2:**
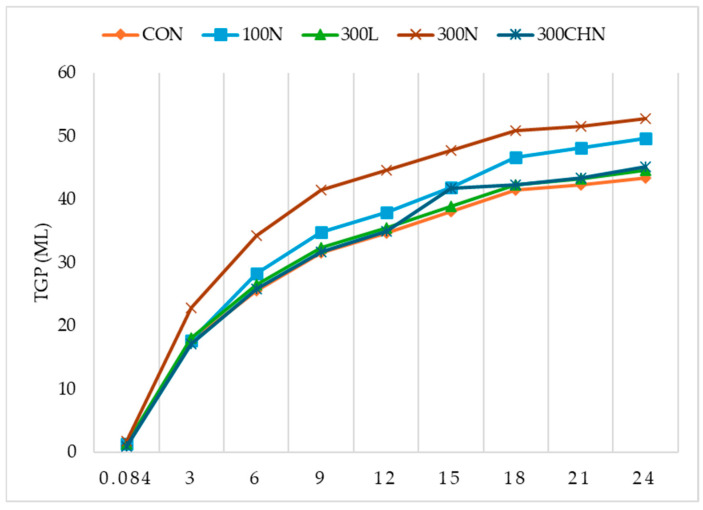
Curve behavior of total gas production of treatments during fermentation times.

Table 5 presents the results of the polynomial regression analysis of total gas production for the treatments over time. The treatments showed different curve behaviors over time, as shown in Figure 2. The treatments with the best cubic fits were the CON and 300N treatments. For the rest of the treatments, the best data fits were with the quadratic model.

Total gas production in all treatments was dependent (β_1_; *p* < 0.05) on time, and the model fit was 78.48% for 300N and 85.00% for 300CHN (Table 6). First-order linear regression coefficients for in vitro TGP were different (*p* < 0.05) for β_0_ and β_1_, where 300N presented the highest β_0_ and β_1_ values. The β_0_ coefficient was relatively the lowest for 300CHN, and the lowest (*p* < 0.05) slope (β_1_) was seen with 300L. The lowest linear model fit was for 300N, and the highest was for 100N.

Quadratic analysis (β_1_ and β_11_) revealed that TGP was dependent (*p* < 0.05) on time for treatments, and adjustment of models increased for treatments from 96.37% (300N) to 97.77% (100N) (Table 6). Second-order linear regression coefficients for TGP (mL) were significant (*p* < 0.05) between treatments for slopes (β_1_ and β_11_), where β_1_ was highest in 300N and lowest in CON, while β_11_ was highest in 300CHN and lowest in 300N.

### 3.6. Ruminal Fluid pH and Ammonia (NH_3_)

Table 7 presents the behavior of goat ruminal fluid pH during fermentation of experimental diets for 24 h. The time fixed effect was significant (*p* < 0.05). At 0 h, 300L and 300CHN exhibited the highest pH values, and 300N showed the lowest pH. However, at 3 h, 300N had the highest pH, while 300L had the lowest. At 6 h, a tendency was shown, where 300CHN exhibited the highest pH and 300N had the lowest pH.

The ammonia (NH_3_) during in vitro fermentation of OEO-treated diets in goat ruminal fluid was significant (*p* < 0.05) for treatment–time interaction; likewise, the time effect was significant (*p* < 0.05) as shown in Table 7. At 3 h, the 300CHN group had NH_3_ levels 39.20% lower compared to the CON group, but similar to the CON group at the other times evaluated. At 12 h, 300CHN had 25.16% higher NH_3_ concentrations compared to 100 L, and 300L was 31.51% higher compared to 100 L.

In the quadratic linear regression analysis (β_1_ and β_11_) of treatments, it was found that pH and NH_3_ were dependent (*p* < 0.05) on time during in vitro fermentation. For pH, coefficient β_11_ was significant (*p* < 0.01) for CON, 100N, 300N, and 300CHN. For NH_3_, the coefficient β_11_ was significant (*p* < 0.01) for 300L, 300N, and 300CHN. On the other hand, for pH there was a model fit of 77.79% for 300L and 90.79% for 300N, and for NH_3_, 73.42% for 100N and 97.31% for 300CHN (Table 8). Second-order linear regression coefficients (β_0_, β_1_, and β_11_) for pH were not different (*p* > 0.05). However, NH_3_ was different (*p* < 0.05) for linear (β_1_) and quadratic (β_11_) slopes. For NH_3_, the coefficient β_1_ was highest for 300L and lowest for 100N. In contrast, β_11_ was highest for 100N and lowest for 300L.

First-order linear regression analysis (β_1_) showed that pH and NH_3_ were dependent (*p* < 0.05) on time for treatments. The model fit for pH was between 54.13% (CON) and 65.89% (300CHN). In comparison, NH_3_ was 58.88% (300L) and 91.05% (300N) (Table 8). Y-intercept (β_0_) and slope (β_1_) for treatments were not different (*p* > 0.05) for pH and NH_3_.

## 4. Discussion

### 4.1. Nanoparticle Characterization

Hosseini et al. [14] reported that most of the CHNs and OON in their study were distributed between 40 and 80 nm. However, those authors pointed out that CHNs were smaller than OON and similar to CH nanoparticles obtained in the current study. The increase in nanoparticle size is due to the OEO charge inside chitosan nanoparticles [14], which can explain the range variation found for OON and CHNs. According to Bhatia [28], polymeric nanoparticles must have a particle size of 10–1000 nm. Particles in the current study presented sizes within that range.

On the other hand, smaller nanoparticle sizes and corresponding greater surface areas result in rapid drug release, in contrast to larger nanoparticle sizes and slower drug release [28]. In polymeric nanoparticles for the purpose of drug delivery, like chitosan, the mechanism of degradation is hydrolysis-driven [29]. It is known that in the rumen, polymer-fermenting bacteria can ferment disaccharides and monosaccharides produced from initial hydrolysis; in addition, ruminal fungi produce hydrolytic enzymes, like cellulases, hemicellulases, pectin lyases, amylases, and proteases required to break down the major components of plant biomass [30]. A larger nanoparticle size would ensure a controlled release of oregano oil because there is a greater surface area for the interaction of the nanoparticle wall (chitosan) with microorganisms and enzymes in the ruminal microenvironment before coming in contact with oregano oil.

### 4.2. In Vitro Dry Matter Digestibility

The ivDMD is used to evaluate the nutritional quality of foods [31]. Benchaar et al. [7] examined the effects of OEO (200 mg/L), thymol (400 mg/L), eugenol (800 mg/L), and carvacrol (400 mg/L) on the ruminal fluid of Holstein cows, finding that phenolic compounds had an effect on DMD at 24 h when compared to the control group (31.7%), with percentages of digestibility of 30.5% for thymol, 24.5% for eugenol and 26.8% for carvacrol. These results were lower than those obtained in the present study where OEO (containing carvacrol and thymol) was evaluated, which indicated a better ivDMD when OEO was used in pure form and the nanoparticulated form. Contrary to the current study, Benchaar et al. [7] and Righi et al. [10] reported no interaction effect and did not assess the treatment–time interaction of ivDMD.

Righi et al. [10] evaluated OEO doses at 0.5 mg/L in different TMR substrates (alfalfa hay, soybean meal, and cornmeal). Those authors obtained differences at 24 h for ivDMD, where OEO was lower (61.56%) in TMR as compared to eugenol, thymol, and carvacrol with ivDMD of 70.05, 75.38 and 75.78%, respectively. ivDMD predicts the in vivo behavior of animal production, where low DMD decreases milk production [32]. Hence, the current study presented the highest ivDMD for 300N, which could suggest that this treatment has potential to improve feed efficiency, which could contribute to productive performance. No similar studies have evaluated the OON effect on the ruminal fermentation of ivDMD. However, this work demonstrates 300N improved availability of nutrients since increasing ivDMD at 12 h, 36, and 48. Time at 12 h is relevant since feed retention time in the rumen of goats is 14.43 h [33]. These data help to set a precedent for researchers seeking to implement doses of essential oils using nanoparticles as a vehicle or carrier in goats. It is important to highlight that 100N had a similar effect on digestibility, compared with a higher dose of liquid oregano oil (300L); hence, nanoparticles could be used to have similar effects of liquid OEO with a minor dose. The 300N treatment, although statistically different at 12 h and 48 h compared to the CON treatment and at 36 h against 300L, is numerically superior to all treatments over time. These results suggest that at longer times after 12 h, it could favor fiber digestion, because fibrous feed remains in the rumen for a longer period. This effect is corroborated at 36 h with the ivNDFD_om_ of 300N, since it is lower than the rest of the treatments.

### 4.3. Neutral Detergent Fiber Digestibility

Neutral detergent fiber digestibility measures forage cell wall cellulose and hemicellulose fractions [17]. The effect of OEO on ivNDFD has been reported in a TMR for Holstein cows [7], in which there was a significant difference compared to the control group (23.5%) with a decrease in ivNDFD for OEO (16.9%). Benchaar et al. [7] examined the ivNDFD effects of the OEO compounds carvacrol (12.2%), thymol (21.2%), and eugenol (7.2%), which were different with respect to the control group (27.7%). Benchaar et al. [7] obtained a value of 33%, while Righi et al. [10] obtained 34.8% ivNDFD, compared to 26.60% in the current study. Those results demonstrated that OEO components decreased ivNDFD. In the current study, at 24 h, no difference was found for ivNDFD, but OON decreased ivNDFD_om_. The energy in forages is mainly derived from structural carbohydrates in the cell wall of plants (fiber), and digestibility is also a key determinant of feed nutritive value [34]. These results evidence that liquid oregano oil had less properties to help in structural carbohydrate digestion at 36 h. The compounds in oregano essential oil have a pH-reducing effect on ruminal pH, as demonstrated by the pH results of this study. This affects the activity of cellulolytic bacteria, which are more active at higher pH levels. The digestion rate of NDF will depend on the feed composition and maturity of forages. In this study, the feed consisted of a TMR containing a high non-fiber carbohydrate percentage (40.41%). In a diet containing a higher fiber percentage, 300L could improve NDF digestibility.

Righi et al. [10] reported the highest ivNDFD (72.14%) with OEO at 24 h, followed by eugenol (70.11%), control (69.99%), carvacrol (67.87%), and thymol (62.77%). The ivNDFD_om_ results from the current study with OON are similar to those of Righi et al. [10]. Oregano oil composition in the current study included 65.02% carvacrol. A decrease in ivNDFD_om_ has been reported by other authors [7,35,36] when evaluating essential oils rich in phenolic compounds or pure phenolic compounds, suggesting that fibrolytic bacteria can be sensitive to these compounds.

### 4.4. First- and Second-Order Linear Regression Analysis of Digestibility

The regression coefficients were not different, indicating that OEO levels and offering form did not influence a change in ivDMD and ivNDFD_om_ during 48 h of fermentation. Similar studies [7,35,36] did not evaluate linear regression coefficients of ivDMD and ivNDFD_om_ of goat rations treated with liquid and nanoparticulated OEO. Slope analysis did not show differences in both variables; this result reflected that levels and the method of presentation of OEO did not influence quadratic regression results and a change in ivDMD and ivNDFD_om_. In quadratic-order coefficients, R^2^ was higher for all treatments, indicating there is an inflection point in the dose used over time. This suggests that the doses used in this study could be adjusted to a slightly lower dose to avoid reaching the inflection point by inhibiting dry matter digestibility.

### 4.5. Total Gas Production (TGP)

Goiri et al. [37] evaluated the effects of different degrees of CH acetylation and monensin during in vitro sheep rumen digestion and fermentation of maize silage, highlighting that CH addition caused a reduction in gas production 6–10 h post-inoculation. Treatments in the current study increased the TGP as time increased, which was corroborated by first-order regression analysis. While Zhou et al. [11] reported a TGP of 128.9 mL at 24 h at a dose of 130 ppm of OEO, results from the present investigation indicated 48.74 mL of TGP for the 100N treatment. This difference in the volume of gas produced may be due to the CH interaction with OEO and due to the ability of CH to reduce the TGP [37].

Macheboeuf et al. [8] evaluated OEO dose (0, 1.5, 3.0, 5.0, and 10.0 mmol/L) effects and three pure constituents (thymol, carvacrol, and cinnamaldehyde) added to a substrate and incubated for 5 and 16 h on in vitro ruminal fermentation with fluid collected from Texel sheep. Those authors found that carvacrol presented a lower TGP with 5.0 mmol/L OEO added in the substrate (0.423 mmol/L at 5 h and 0.536 mmol/L at 16 h) compared to TGP in the control with 0 mmol/L OEO added (1.663 mmol/L at 5 h and 3.815 mmol/L at 16 h), showing that OEO decreased TGP. At the times and concentrations used by those authors, linear, quadratic, and cubic effects were also observed. Macheboeuf et al. [8] observed similar effects on TGP at 16 h using thymol to those in the current study at 15 h of fermentation with OEO in goat ruminal fluid. In the current study, no difference was obtained between treatments at 5 min, contrary to the results of Macheboeuf et al. [8], while, at 15 h, 300 ppm of chitosan nanoparticle-encapsulated liquid oregano oil was the treatment with the highest TGP, which suggests that OON benefited ruminal fermentation.

Results showed that 300N increased TGP. Zhou et al. [11] studied the effects of different levels of OEO inclusion (0, 13, 52, 91, and 130 mg/L) in a 65.5:34.5 forage-to-concentrate ratio in the ruminal fluid of Merino × Criollo sheep. Those authors reported that TGP per 24 h at 0 and 130 mg/L (137 and 128.9 mL of TGP, respectively) was greater following quadratic analysis than that of 13 (124.4 mL), 52 (124.2 mL), and 91 (125 mL) mg/L. Zhou et al. [11] reported that as OEO concentration increased, TGP decreased. This observation contrasts with 100N and 300N of the current study, since the higher the dose of OON, the higher the fermentation, which coincides with results of Macheboeuf et al. [8]. Zhou et al. [11] observed that EO type and dose can lower TGP and reduce methane gas production, suggesting that OEO inhibits methane gas synthesis. Gas released from feeds inoculated with rumen fluid reflects microbial activity [38]. Higher TGP indicates that rumen microbes were able to ferment feed better due to a higher content of fermentable substrate, increasing nutrient availability and leading to energy supply via short-chain fatty acid production [39]. The results obtained with OEO and OON suggested that chitosan nanoparticle formulations of phenolic compounds can have a positive effect on TGP due to the greater fermentative activity. This can be proved based on the results of ivDMD, where at 12 h, the 300N group was superior to the CON group; it can be observed that at this same time, no difference was found in the digestion of ivNDFD_om_, so it is inferred that 300N promotes the specific digestion of non-structural carbohydrates, since at this time the NH_3_ concentrations were similar between CON and 300N, demonstrating that the effect of 300N is specific on non-structural carbohydrates and not on the protein of the feed. Results of linear regression coefficients (β_0_ and β_1_) for TGP with 300N indicated that fermentation started at a higher point (β_0_). Thus, this treatment obtained the highest slope (β_1_). In quadratic regression coefficients, it can be observed that 300N obtained the highest inflection point in the curve compared to the other treatments, indicating that this treatment can increase fermentation over time, suggesting greater activity of ruminal bacteria.

The results of the nonlinear polynomial regression analysis of total gas production showed that most treatments were best fitted to the quadratic model. This result is interesting because it demonstrates that there is a point at which the maximum TGP level is reached, and then the TGP level decreases. For in vitro studies, this type of behavior is expected because there is no natural gas escape, as occurs in an in vivo study. This study provides a first general overview of what is happening at the gas production level. However, it has some limitations, including the calculation of variables to investigate the kinetics of in vitro gas fermentation (inflection point, maximum rate of gas production, and lag phase), which could help elucidate the processes that occur during fiber fermentation. To better understand these variables, an experimental design designed exclusively for these parameters would be necessary. Likewise, future studies could evaluate the aforementioned variables using a quadratic fit of the data, which would provide more detailed information to help predict potential changes in ruminal fermentation. Future studies could also focus on comparing whether the quadratic effect found on the in vitro data is also found in vivo or whether the in vivo fit to the data is linear due to the natural ways in which gas escapes from the interior of the animal, such as via belching and intestinal gas, or whether oregano oil (alone or in nanoparticle form) modifies TGP. The treatments that best fit the quadratic model were those containing both chitosan and oregano components (nanoparticles). The treatments that best fit the quadratic model were those containing oregano oil and chitosan separately, which best fit the cubic model. These results indicate a combined effect on TGP when the components are in nanoparticles compared to the individual components, indicating that fermentation increases again after decreasing for the chitosan and oregano treatments alone. These results indicate that the encapsulation of oregano oil in chitosan modifies the release of its components over time, causing different behaviors in the TGP curve.

### 4.6. Ruminal pH and Ammonia (NH_3_)

At 3 h, the 300L treatment obtained a significantly lower pH than the other treatments, which could reduce bacterial activity at the beginning of ruminal fermentation because cellulolytic enzyme-producing bacteria have higher activity at pH levels close to 6. This effect could be due to the lack of an encapsulating material (chitosan), as the other treatments were similar and shared chitosan as a common encapsulating material. Results from this study at 24 h of fermentation indicated that 300N lowered pH more than other treatments. Cardozo et al. [40] mentioned that variability in EO activity in ruminal fermentation can be affected by ruminal pH, which can influence dissociated or undissociated states of EO molecules, since the undissociated hydrophobic form of EO molecules appears to have higher antimicrobial activity than dissociated EO components [9,40]. This suggests that 300N encased in the chitosan nanoparticles was not dissociated, causing greater antimicrobial activity and loss of fibrolytic bacteria sensitive to low pH [41]. Also, Ultee et al. [42] examined the importance of the phenolic hydroxyl group of carvacrol as essential for action against *Bacillus cereus*. Those authors indicated that given a pKa of phenolic compounds of 10, 0.1% of the carvacrol hydroxyl groups would be dissociated at a pH under 7. The OEO used in the current study was 65% carvacrol. The hydroxyl group of this compound and the presence of its system of delocalized electrons are important for the antimicrobial activity of carvacrol.

Ammonia concentration has been used as an indicator of in vivo and in vitro microbial protein degradation, as well as the utilization of non-protein nitrogen [43]. According to Zhou et al. [11], EO are known to reduce NH_3_ and inhibit protein deamination. Other studies indicated that different types and doses of EO can inhibit NH_3_ concentrations [11,40,44,45]. Benchaar et al. [7] reported NH_3_ concentrations at 24 h in the in vitro rumen microbial fermentation experiment groups of 11.7 mM (control), 10.1 mM (carvacrol), 12.0 mM (thymol), 8.4 mM (eugenol) and 13.2 mM (oregano). Those authors did not obtain any difference at 24 h, which is similar to what was reported in the current study with OEO and OON. Macheboeuf et al. [8] reported that an OEO dosage of 1.5 mmol/L of ruminal fluid yielded a higher production of NH_3_ (0.380 mmol) and a lower production (0.130 NH_3_) for the OEO dosage of 5.0 mmol/L of ruminal fluid. In the current study, at 12 h, 100N had the lowest NH_3_ content, indicating less microbial protein degradation. Zhou et al. [11] found linear and quadratic effects, reporting that treatments with the highest NH_3_ production were the control (15.8 NH_3_) and 13 mg/L OEO (15.6 NH_3_). In the present study, 300CHN exhibited less NH_3_ at 3 h, indicating that CHNs can inhibit the degradation of microbial protein in vitro, potentially increasing its arrival to the small intestine. The results of the first-order linear regression indicate that the model fits better for the 300N treatment, explaining 91.05% of NH_3_ data variability compared to other treatments, which indicates a good model fit. In the quadratic regression analysis, it can be observed that the 300N and 300L treatments obtained the highest inflection point, indicating a higher amount of NH_3_ for a longer time. This effect is beneficial in terms of milk production. However, the CHN treatment behaved similarly to 300N over time, suggesting that chitosan has an effect on protein digestion in the rumen.

## 5. Conclusions

In this study, the use of OEO on in vitro ruminal fermentation parameters was explored, as well as the use of nanoparticles with biodegradable polymers (chitosan) to improve the ruminal efficiency of OEO. The in vitro results have some limitations and need to be tested under real production conditions to investigate the in vivo effects of the components of nanoparticulate oregano oil, especially 300N, which obtained better results for in vitro ruminal fermentation. Future studies could evaluate the use of nanoparticulated oregano oil in goat feed on ruminal health, ruminal and intestinal microbial populations, growth, milk production, and meat quality to generate information that helps to establish optimal doses according to the animal’s productive purpose. The objective of this study was to evaluate in vitro ruminal fermentation parameters, and it was demonstrated that 300N performed equally or better than liquid oregano oil (without nanoparticles) in most vitro ruminal fermentation parameters. The information presented is helpful for understanding the role of OEO at the ruminal level, since 300N improved dry matter digestion, suggesting greater nutrient availability and a possible improvement in carbohydrate digestion. Furthermore, the linear and nonlinear regression models used facilitate a better understanding of the fermentation behavior of diets with OEO in nanoparticles or liquid, establishing that, for the TGP, the cubic and quadratic models provided a better fit than the linear model.

## Figures and Tables

**Table 1:** Experimental diet formulated for goats.

Ingredients	Percentage
Alfalfa pellet	30.00
Soy husk	6.00
Ground corn	31.70
Wheat barn	8.00
Soybean paste	8.20
Cotton seed	10.00
Molasses	4.00
Experimental supplement ^1^	0.10
Vitamins and minerals mix	2.00
Chemical composition	Percentage DM ^2^
Moisture	11.60
ME (kcal/kg)	2.64
NE_L_ (kcal/kg)	1.71
CP	16.48
RUP	5.73
NDF	31.34
ADF	21.51
peNDF	60.18
EE	4.39
Ash	6.87
NFC	40.41
Minerals (%) ^3^	
Ca	0.80
P	0.51
K	1.25

^1^ Experimental supplement represents the treatment ingredients added to the diet formulation for goats. ^2^ DM, dry matter; ME, metabolizable energy; NE_L_, net energy lactation; CP, crude protein; RUP, rumen undegradable protein; NDF, neutral detergent fiber; ADF, acid detergent fiber; peNDF. physically effective neutral detergent fiber; EE, ether extract; NFC, non-fiber carbohydrates. ^3^ Ca, calcium; P, phosphorus; K, potassium.

**Table 2:** Percentage of in vitro dry matter and in vitro neutral detergent fiber (organic matter basis) digestibility of diets treated with oregano oil and fermented in goat ruminal fluid.

Variables/Treatments ^†^	Time (h)					*p*-Value ^§^		
	0	12	24	36	48	T_i_	δ_j_	(Tδ)_ij_
ivDMD								
CON	8.08	37.12 ^b^	47.44	52.79 ^ab^	55.73 ^b^	0.001	0.001	0.723
100N	7.99	37.96 ^ab^	47.27	53.07 ^ab^	56.88 ^ab^			
300L	8.22	37.94 ^ab^	46.74	52.27 ^b^	57.25 ^ab^			
300N	8.40	39.33 ^a^	47.72	54.40 ^a^	57.99 ^a^			
300CHN	8.32	38.26 ^ab^	47.56	53.22 ^ab^	57.16 ^ab^			
SEM	0.41	0.35	0.56	0.47	0.48			
*p*-value	0.954	0.002	0.774	0.036	0.034			
ivNDFD_om_								
CON	35.38	34.2	29.79	27.57 ^ab^	26.72	0.437	0.001	0.926
100N	34.94	32.06	28.95	27.16 ^ab^	25.76			
300L	35.23	32.01	30.13	28.39 ^a^	25.79			
300N	35.41	33.37	28.83	26.39 ^b^	24.86			
300CHN	34.53	30.86	29.57	27.99 ^b^	25.77			
SEM	0.79	1.63	0.42	0.41	0.48			
*p*-value	0.93	0.65	0.16	0.014	0.136			

^†^ ivDMD, in vitro dry matter digestibility; ivNDF_om_, in vitro digestibility of neutral detergent fiber (organic matter basis) digestibility; CON, diet without oregano oil or nanoparticles; 100N, diet with 100 ppm of oregano oil in nanoparticles; 300L, diet with 300 ppm of liquid oregano oil; 300N, diet with 300 ppm of oregano oil in nanoparticles; 300CHN, diet with 300 ppm of empty nanoparticles. ^§^ T_i_, effect of treatment; δ_j_, effect of evaluation time; (Tδ)_ij_, interaction effect between treatment and evaluation time; SEM, standard error of the mean. ^a,b^ Means in the same column and with different letters show significant differences between treatments at each time (*p* < 0.05).

**Table 3:** First- and quadratic-order regression coefficients of in vitro digestibility of diets treated with oregano oil and fermented in goat ruminal fluid.

Variable/Treatments ^†^	First-Order Coefficients				Quadratic-Order Coefficients ^§^				*p*-Value	
	β_0_	β_1_	R^2^	*p*-Value (β_1_)	β_0_	β_1_	β_11_	R^2^	β_1_	β_11_
ivDMD										
CON	18.04	0.9247	81.90	0.001	9.87	2.29	−0.02836	97.45	0.001	0.001
100N	18.06	0.9407	82.32	0.001	10.08	2.27	−0.02770	96.79	0.001	0.001
300L	18.71	0.9523	82.34	0.001	10.66	2.30	−0.02797	96.87	0.001	0.001
300N	18.01	0.9367	83.60	0.001	10.47	2.19	−0.02617	96.86	0.001	0.001
300CHN	18.37	0.9390	82.53	0.001	10.42	2.26	−0.02760	97.10	0.001	0.001
SEM	0.85	0.02			0.46	0.04	0.001			
*p*-value	0.809	0.910			0.754	0.367	0.162			
ivNDFD_om_										
CON	35.52	−0.1997	53.11	0.001	35.93	−0.2670	0.00141	53.64	0.012	0.491
100N	34.43	−0.1938	69.52	0.001	35.03	−0.2953	0.00212	71.19	0.001	0.126
300L	35.39	−0.2341	42.36	0.001	35.83	−0.3080	0.00154	42.73	0.044	0.605
300N	34.81	−0.1876	71.85	0.001	35.01	−0.2206	0.00069	72.05	0.001	0.591
300CHN	33.82	−0.1700	65.45	0.001	34.2	−0.2323	0.00130	66.22	0.001	0.334
SEM	0.82	0.02			0.93	0.07	0.001			
*p*-value	0.593	0.424			0.687	0.890	0.957			

^†^ CON, diet without oregano oil or nanoparticles; 100N, diet with 100 ppm of oregano oil in nanoparticles; 300L, diet with 300 ppm of liquid oregano oil; 300N, diet with 300 ppm of oregano oil in nanoparticles; 300CHN, diet with 300 ppm of empty nanoparticles; SEM, standard error of the mean; ivDMD, in vitro dry matter digestibility; ivNDFD_om_, in vitro neutral detergent fiber on an organic matter basis. ^§^ β_0_, the *x*-axis intercept when *Χ* = 0; β_1_, linear regression coefficient, which indicates changes in *y* when the value of *Χ* is increased by one unit; β_11_, quadratic regression coefficient, which indicates changes in *y* when the value of *Χ* is increased by one quadratic unit; R^2^, the coefficient of determination.

**Table 4:** Milliliters of total gas production during in vitro fermentation in goat ruminal fluid of diets treated with oregano oil.

Treatments ^†^	Time (h) ^1^								
	0.084	3	6	9	12	15	18	21	24
CON	1.15	17.50 ^b^	25.66 ^b^	31.62 ^b^	34.66 ^b^	38.12 ^b^	41.47 ^b^	42.31 ^b^	43.46 ^b^
100N	1.36	16.05 ^b^	27.38 ^b^	33.97 ^b^	37.11 ^b^	41.04 ^b^	45.75 ^ab^	47.32 ^ab^	48.74 ^ab^
300L	1.36	18.12 ^ab^	26.60 ^b^	32.36 ^b^	35.60 ^b^	38.95 ^b^	42.41 ^b^	43.25 ^b^	44.61 ^ab^
300N	1.78	21.87 ^a^	33.31 ^a^	40.47 ^a^	43.61 ^a^	46.75 ^a^	49.90 ^a^	50.65 ^a^	51.78 ^a^
300CHN	1.05	17.33 ^b^	26.00 ^b^	31.78 ^b^	35.18 ^b^	38.69 ^b^	42.46 ^b^	43.59 ^b^	45.35 ^ab^
SEM	0.19	1.04	0.86	0.98	0.96	1.12	1.37	1.48	1.77
*p*-value	0.103	0.023	0.001	0.001	0.001	0.001	0.003	0.007	0.036
				T_i_	δ_j_	(Tδ)_ij_			
				0.001	0.001	0.928			

^†^ CON, diet without oregano oil or nanoparticles; 100N, diet with 100 ppm of oregano oil in nanoparticles; 300L, diet with 300 ppm of liquid oregano oil; 300N, diet with 300 ppm of oregano oil in nanoparticles; 300CHN, diet with 300 ppm of empty nanoparticles. ^1^ T_i_, effect of treatment; δ_j_, effect of evaluation time; (Tδ)_ij_, interaction effect between treatment and evaluation time; SEM, standard error of the mean. ^a,b^ Means in the same column with different letters show significant differences between treatments at each time (*p* < 0.05). ^1^ 0.084 h is equivalent to 5 min; the milliliters of total gas production is per gram of incubated dry matter.

**Table 5:** Polynomial regression analysis of total gas production per treatment over time.

	Regression Equation ^2^				SE	R^2^ (%)	*p*-Value
Treatment ^1^	β_0_	β_1_	β_2_	β_3_			
Linear							
CON	11.880	+1.565	-	-	5.83	84.70	0.000
100N	10.500	+1.923	-	-	8.33	78.20	0.000
300L	12.340	+1.594	-	-	8.87	66.70	0.000
300N	15.610	+1.895	-	-	10.07	69.50	0.000
300CHN	10.700	+1.686	-	-	10.36	63.00	0.000
Quadratic							
CON	4.176	+3.746	−0.091	-	2.42	97.70	0.001
100N	3.541	+4.200	−0.098	-	6.49	87.10	0.000
300L	4.511	+3.810	−0.092	-	7.46	76.90	0.000
300N	5.408	+4.953	−0.129	-	7.33	84.20	0.000
300CHN	3.635	+3.805	−0.089	-	9.26	71.10	0.001
Cubic							
CON	1.794	+5.397	−0.272	+0.005	1.40	99.40	0.015
100N	1.488	+5.882	−0.288	+0.005	6.29	88.20	0.081
300L	1.987	+5.559	−0.284	+0.005	7.31	78.30	0.079
300N	2.101	+7.466	−0.409	+0.008	6.89	86.40	0.013
300CHN	1.438	+5.474	−0.276	+0.005	9.19	72.20	0.203

^1^ CON, diet without oregano oil or nanoparticles; 100N, diet with 100 ppm of oregano oil in nanoparticles; 300L, diet with 300 ppm of liquid oregano oil; 300N, diet with 300 ppm of oregano oil in nanoparticles; 300CHN, diet with 300 ppm of empty nanoparticles. ^2^ β_0_, the *x*-axis intercept when *Χ* = 0; β_1_, linear regression coefficient, which indicates changes in *y* when the value of *Χ* is increased by one unit; β_2_, quadratic regression coefficient, which indicates changes in *y* when the value of *Χ* is increased by one quadratic unit; β_3_, cubic regression coefficient, which indicates changes in *y* when the value of *Χ* is increased by one cubic unit; R^2^, the coefficient of determination.

**Table 6:** First and quadratic order regression coefficients of total gas production from diets treated with oregano oil following in vitro fermentation in goat ruminal fluid.

Treatments ^†^	First-Order Coefficients				Quadratic-Order Coefficients ^§^				*p*-Value	
	β_0_	β_1_	R^2^	*p*-Value (β_1_)	β_0_	β_1_	β_11_	R^2^	β_1_	β_11_
CON	11.88 ^b^	1.56 ^ab^	84.15	0.001	4.18	3.75 ^b^	−0.09067 ^a^	97.45	0.001	0.001
100N	12.80 ^b^	1.72 ^ab^	87.14	0.001	4.58	4.04 ^b^	−0.09674 ^a^	97.77	0.001	0.001
300L	12.34 ^b^	1.55 ^b^	83.98	0.001	4.51	3.81 ^b^	−0.09215 ^a^	97.41	0.001	0.001
300N	15.99 ^a^	1.81 ^a^	78.48	0.001	5.68	4.73 ^a^	−0.12146 ^b^	96.37	0.001	0.001
300CHN	11.41 ^b^	1.59 ^ab^	85.00	0.001	3.89	3.78 ^b^	−0.08865 ^a^	97.11	0.001	0.001
SEM	0.55	0.06			0.44	0.09	0.004			
*p*-value	0.001	0.030			0.103	0.001	0.001			

^†^ CON, diet without oregano oil or nanoparticles; 100N, diet with 100 ppm of oregano oil in nanoparticles; 300L, diet with 300 ppm of liquid oregano oil; 300N, diet with 300 ppm of oregano oil in nanoparticles; 300CHN, diet with 300 ppm of empty nanoparticles; SEM, standard error of the mean. ^§^ β_0_, the *x*-axis intercept when *Χ* = 0; β_1_, linear regression coefficient, which indicates changes in *y* when the value of *Χ* is increased by one unit; β_11_, quadratic regression coefficient, which indicates changes in *y* when the value of *Χ* is increased by one quadratic unit; R^2^, the coefficient of determination. ^a,b^ Means in the same column and with different letters show significant differences between treatments at each time (*p* < 0.05).

**Table 7:** Ruminal fluid pH and ammonia ^1^ evaluation in feed treated with oregano oil and fermented in vitro in goat ruminal fluid.

Variables/Treatments ^†^	Time (h)					*p*-Value ^§^		
	0	3	6	12	24	T_i_	δ_j_	(Tδ)_ij_
pH								
CON	5.74 ^ab^	5.11 ^bc^	5.15 ^ab^	4.85	4.86	0.483	0.001	0.543
100N	5.72 ^ab^	5.12 ^b^	5.14 ^ab^	4.86	4.86			
300L	5.77 ^a^	5.08 ^c^	5.15 ^ab^	5.02	4.74			
300N	5.67 ^b^	5.24 ^a^	5.13 ^b^	4.66	4.86			
300CHN	5.75 ^a^	5.21 ^a^	5.19 ^a^	4.98	4.88			
SEM	0.02	0.01	0.01	0.16	0.08			
*p*-value	0.015	0.001	0.055	0.585	0.676			
NH_3_								
CON	0.52	1.25 ^a^	1.01	1.43 ^ab^	1.61	0.236	0.001	0.001
100N	0.54	1.18 ^ab^	1.02	1.13 ^b^	1.59			
300L	0.51	1.09 ^abc^	1.06	1.65 ^a^	1.50			
300N	0.61	0.87 ^bc^	0.96	1.46 ^ab^	1.72			
300CHN	0.61	0.76 ^c^	1.1	1.51 ^a^	1.50			
SEM	0.05	0.07	0.04	0.08	0.07			
*p*-value	0.415	0.004	0.142	0.012	0.264			

^1^ pH, potential of hydrogen; NH_3,_ ammonia in mg/Dl. ^†^ CON, diet without oregano oil or nanoparticles; 100N, diet with 100 ppm of oregano oil in nanoparticles; 300L, diet with 300 ppm of liquid oregano oil; 300N, diet with 300 ppm of oregano oil in nanoparticles; 300CHN, diet with 300 ppm of empty nanoparticles. ^§^ T_i_, effect of treatment; δ_j_, effect of evaluation time; (Tδ)_ij,_ interaction effect between treatment and evaluation time; SEM, standard error of the mean. ^a–c^ Means in the same column and with different letters show significant differences between treatments at each time (*p* < 0.05).

**Table 8:** First- and quadratic-order regression coefficients for pH and NH_3_ during in vitro ruminal fermentation in goat ruminal fluid of diets treated with oregano oil.

Variable/Treatments ^†^	Coefficients				Coefficients ^§^				*p*-Value	
	β_0_	β_1_	R^2^	*p*-Value (β_1_)	β_0_	β_1_	β_11_	R^2^	β_1_	β_11_
pH										
CON	5.40	−0.0286	54.13	0.003	5.62	−0.106	0.00313	82.82	0.001	0.002
100N	5.39	−0.0282	57.31	0.001	5.61	−0.1031	0.00303	87.24	0.001	0.001
300L	5.45	−0.0327	61.62	0.001	5.59	−0.0826	0.00202	77.79	0.006	0.06
300N	5.38	−0.0303	58.77	0.009	5.65	−0.1255	0.00385	90.79	0.001	0.004
300CHN	5.46	−0.0290	65.89	0.001	5.64	−0.0914	0.00252	89.05	0.001	0.001
SEM	0.03	0.004			0.04	0.02	0.001			
*p*-value	0.208	0.887			0.813	0.731	0.683			
NH_3_										
CON	0.8300	0.03701	67.19	0.001	0.6720	0.0931 ^ab^	−0.00227 ^abc^	79.06	0.005	0.054
100N	0.7833	0.03430	70.48	0.001	0.7297	0.0533 ^b^	−0.00077 ^a^	73.42	0.051	0.44
300L	0.8350	0.03640	58.88	0.001	0.5583	0.1345 ^a^	−0.00396 ^c^	91.42	0.001	0.001
300N	0.7080	0.04614	91.05	0.001	0.5914	0.0875 ^ab^	−0.00167 ^ab^	96.5	0.001	0.01
300CHN	0.7550	0.03793	74.69	0.001	0.5442	0.1127 ^a^	−0.00302 ^bc^	97.31	0.001	0.001
SEM	0.04	0.004			0.05	0.01	0.0004			
*p*-value	0.294	0.310			0.072	0.007	0.004			

^†^ CON, diet without oregano oil or nanoparticles; 100N, diet with 100 ppm of oregano oil in nanoparticles; 300L, diet with 300 ppm of liquid oregano oil; 300N, diet with 300 ppm of oregano oil in nanoparticles; 300CHN, diet with 300 ppm of empty nanoparticles; SEM, standard error of the mean. ^§^ β_0_, the *x*-axis intercept when *Χ* = 0; β_1_, linear regression coefficient, which indicates changes in *y* when the value of *Χ* is increased by one unit; β_11_, quadratic regression coefficient, which indicates changes in *y* when the value of *Χ* is increased by one quadratic unit; R^2^, the coefficient of determination. ^a–c^ Means in the same column and with different letters show significant differences between treatments at each time (*p* < 0.05).

## Data Availability

The entire dataset supporting this study’s results is available upon request to the corresponding author, Jocelyn Cyan López-Puga. However, the dataset is not publicly available due to the privacy of research participants.

## Abbreviations

The following abbreviations are used in this manuscript:

CON	diet with 100 ppm of oregano oil in nanoparticles
300L	diet with 300 ppm of liquid oregano oil
300N	diet with 300 ppm of oregano oil in nanoparticles
300CHN	diet with 300 ppm of empty nanoparticles
ivDMD	in vitro dry matter digestibility
ivNDFD_om_	in vitro neutral detergent fiber digestibility in organic matter basis
TGP	total gas production
pH	potential of hydrogen
NH_3_	ammonia
β	regression coefficient
β_1_	expected change in the dependent variable for a one-unit increase in the independent variable
EO	essential oil
OEO	oregano essential oil
OON	nanoparticle-encapsulated oregano oil
CHN	chitosan nanoparticle
TPP	sodium tripolyphosphate
TMR	total mixed ration
ppm	parts per million
CO_2_	carbon dioxide
GLM	general lineal model
